# Association of miR-196a2 rs11614913 and miR-499 rs3746444 polymorphisms with cancer risk: a meta-analysis

**DOI:** 10.18632/oncotarget.22547

**Published:** 2017-11-20

**Authors:** Wanjun Yan, Xiaoyan Gao, Shuqun Zhang

**Affiliations:** ^1^ Department of Oncology, The Second Affiliated Hospital of Xi’an Jiaotong University, Xi’an, Shaanxi 710004, P.R. China

**Keywords:** miRNA, single nucleotide polymorphism, cancer susceptibility, meta-analysis

## Abstract

**Background:**

MicroRNAs (miRNAs) are small non-coding RNA molecules, which participate in diverse biological processes and may regulate tumor suppressor genes or oncogenes. Rs11614913 in miR-196a2 and rs3746444 in miR-499 are shown to associate with increased/decreased cancer risk. This meta-analysis was performed to systematically assess the overall association.

**Materials and Methods:**

We searched Pubmed, Web of Knowledge, EMBASE, Chinese National Knowledge Infrastructure (CNKI) databases until December 2016 to identify eligible studies. Odds ratios (ORs) and 95% confidence intervals (CIs) were used to estimate the strength of the associations.

**Results:**

We assessed published studies of the association between these microRNA polymorphisms and cancer risk from 56 studies with 21958/26436 cases/controls for miR-196a2 and from 37 studies with 13759/17946 cases/controls for miR-499. The results demonstrated that miR-196a2 rs11614913 was significantly associated with a decreased cancer risk, in particular with a decreased risk for colorectal cancer and gastric cancer, or for Asian population subgroup. In addition, miR-499 rs3746444 polymorphism was observed as a risk factor for cancers, in particular, for breast cancer, or for in the Asian population.

**Conclusions:**

Our meta-analysis suggests that the rs11614913 most likely contributes to decreased susceptibility to cancer, especially in Asians and colorectal cancer and gastric cancer, and that the rs3746444 may increase risk for cancer. Furthermore, more well-designed studies with large sample size are still necessary to further elucidate the association between polymorphisms and different kinds of cancers risk.

## INTRODUCTION

Cancer is reportedly one of the major causes of death among human worldwide [1]. According to GLOBOCAN 2012 report, there were 14.1 million new cases and 8.2 million deaths in 2012 [2]. Recently reported, as a very complex genetic disease, the mechanism of cancer has not been completely elucidated. Moreover, studies have suggested that cancer development results from gene-environment interactions [3].

MicroRNAs (miRNAs) are a class of endogenous small single-stranded, long non-coding RNA molecules, which play critical roles in a extensive range of biologic and pathologic processes, especially in carcinogenesis [4–5]. Accumulating studies indicates that a single miRNA cantarget 200 genes, and approximately 20% of human genes are regulated by the mature miRNA molecules [6]. More than half of miRNAs genes are located in cancer-related genomic regions, indicating that these miRNAs may play a more important key role in the etiology, tumorigenesis, development and prognosis of human cancers than previous research [7].

Single nucleotide polymorphisms (SNPs) occurring in the miRNA gene region may influence the function of specific miRNA molecules and the genetic variation, which are associated with cancer susceptibility through altering miRNA molecules expression [8–9]. Recently, miR-196a2C.T (rs11614913) and miR-499 A.G (rs3746444) have been reported to demonstrate the association with malignant tumors susceptibility [10–17]. For instance, Min et al. [11] demonstrates that the miRNA variants could affect the development of colorectal cancer in the Korean, while Georgeh et al. [17]. showed miR-196a2C.T (rs11614913)and miR-499 A.G (rs3746444) revealed significant risk for developing prostate cancer in North Indian. However, the consequences of these relevant studies remain incomprehensive and controversial. To the best of our knowledge, there is no systematic and comprehensive reports or studies regarding the impact of miR196a2 and miR-499 variants on overall cancer risk in world wide population. Hence, we performed a meta-analysis to clarify the associaton between the miR-196a2C.T (rs11614913)and miR-499 A.G (rs3746444) polymorphisms with cancer susceptibility.

## MATERIALS AND METHODS

### Publication search

We carried out a search in PubMed, ISI Web of Knowledge, EMBASE, Chinese National Knowledge Infrastructure(CNKI) databases for all relevant reports using the key words “microRNA 192” OR “microRNA-192” OR “miR-192” OR “rs11614913” OR “microRNA 499” OR “microRNA-499” OR “miR-499” OR “ rs3746444”) AND (“polymorphism” OR “SNP” OR “variation” OR “locus” OR “mutation”) AND (“cancer” OR “tumor” OR “malignancy” OR “carcinoma” OR “neoplasm”(updated to Dec 30, 2016). The search was limited to English language papers and human subject studies. We evaluated potentially relevant publications by examining their titles and abstracts, thereafter all studies matching the eligible inclusion criteria were retrieved.

### Selection criteria

The following criteria were used to select studies for further meta-analysis: (a) about the miR-196a2 rs11614913/miR-499 rs3746444 polymorphisms and cancer risk, (b) full-text study, (c) from a case-control designed study, (d) genotype frequencies available, (e) sufficient published data for estimating an odds ratio (OR) with 95% confidence interval (CI).

Accordingly, the following exclusion criteria were also used: (a) the design of the experiments were not case-control studies; (b) the source of cases and controls, and other essential information were not provided; (c) reviews and duplicated publications.

### Data extraction

All data were independently abstracted in duplicate by two investigators (Yan and Zhang) using a standard protocol and data-collection form according to the inclusion criteria listed above. The following information was sought from each publication: the first author’s name, year of publication, country of origin, ethnicity, cancer type, source of control (population- or hospital-based controls), genotyping method and number of cases and controls miR-196a2 C/T and/or miR-499 G/A genotypes, respectively (Table 1). Different ethnicity descents were categorized as Caucasian and Asian.

**Table 1:** Summary of published studies included

	Author	Year	Race	Cancer type	Control	Method	Case/control	Polymorphism site
1	Ahn [22]	2012	Asian	gastric cancer	PB	PCR–RFLP	461/477	rs11614913, rs3746444
2	Alshatwi [23]	2012	Asian	Breast Cancer	PB	PCR–RFLP	89/100	rs11614913, rs3746444
3	B.Zhou [24]	2011	Asian	Cervical Squamous Cell Carcinoma	PB	PCR–RFLP	226/309	rs11614913, rs3746444
4	Bansal [25]	2014	Asian	breast cancer	PB	PCR–RFLP	121/164	rs11614913, rs3746444
5	Behnaz [26]	2016	Caucasian	HCC	PB	PCR	103/432	rs11614913
6	Brajušković [27]	2015	Caucasian	prostate cancer	PB	PCR–RFLP	355/312	rs11614913, rs3746444
7	Catucci [28]	2012	Caucasian	breast cancer	PB	PCR–RFLP	1894/2760	rs11614913, rs3746444
8	cheng [29]	2015	Asian	gastric cancer	HB	MassARRAY	363/969	rs3746444
9	Chu [30]	2012	Asian	Oral Cancer	PB	PCR–RFLP	470/425	rs11614913, rs3746444
10	D. Li [31]	2015	Asian	HCC	PB	RT-PCR	184/184	rs3746444
11	Dai [32]	2016	Asian	breast cancer	HB	MassARRAY	560/583	rs11614913, rs3746444
12	Deng [33]	2015	Asian	bladder cancer	PB	PCR–RFLP	159/298	rs11614913, rs3746444
13	Dominik [34]	2011	Caucasian	Breast Cancer	PB	PCR–RFLP	187/171	rs11614913
14	Dou [35]	2010	Asian	glioma	PB	PCR–RFLP	643/656	rs11614913
15	Eman [36]	2016	Caucasian	Hepatic/Renal Cancer	PB	RT-PCR	65/150	rs11614913, rs3746444
16	Gu [37]	2013	Asian	esophageal cancer	PB	PCR–RFLP	380/380	rs11614913, rs3746444
17	H. Chen [38]	2011	Asian	colorectal cancer	HB	PCR–LDR	126/407	rs11614913
18	H. Zhao [39]	2016	Asian	breast cancer	HB	RT-PCR	114/114	rs11614913
19	Hashemi [40]	2016	Asian	prostate cancer	PB	PCR–RFLP	169/182	rs11614913, rs3746444
20	Hikmet [41]	2011	Caucasian	HCC	PB	PCR–RFLP	222/222	rs3746444
21	Hong [42]	2011	Asian	Lung Cancer	HB	PCR–RFLP	406/428	rs11614913
22	Hu [43]	2013	Asian	Glioma	HB	PCR–RFLP	680/690	rs11614913, rs3746444
23	J. Shi [44]	2015	Asian	gastric cancer	PB	RT-PCR	448/452	rs3746444
24	Kim [45]	2012	Asian	colorectal cancer	PB	PCR–RFLP	201/159	rs11614913, rs3746444
25	Kim [46]	2011	Asian	lung cancer	PB	PCR–RFLP	654/640	rs11614913
26	Kou [47]	2014	Asian	HCC	PB	PCR–RFLP	271/532	rs11614913, rs3746444
27	Kshitij [48]	2010	Asian	gallbladder cancer	PB	PCR–RFLP	230/230	rs11614913, rs3746444
28	Kuo [49]	2014	Asian	HCC	PB	PCR–RFLP	188/377	rs11614913, rs3746444
29	Li [50]	2015	Asian	non-Hodgkin lymphoma	PB	RT-PCR	318/320	rs11614913
30	Linhare [51]	2012	Caucasian	Breast cancer	PB	TaqMan	388/388	rs11614913
31	LIU [52]	2010	Asian	HCC	HB	PCR–RFLP	310/222	rs11614913
32	Lv [53]	2013	Asian	colorectal cancer	PB	PCR–RFLP	353/540	rs11614913
33	M. Zhang [54]	2012	Asian	Breast Cancer	PB	PCR–RFLP	252/248	rs11614913
34	Masaaki [55]	2010	Asian	gastric cancer	PB	PCR–RFLP	552/697	rs11614913, rs3746444
35	Min [11]	2012	Asian	Colorectal Cancer	PB	PCR–RFLP	446/502	rs11614913, rs3746444
36	Morales [56]	2016	Caucasian	Breast cancer	PB	TaqMan	440/807	rs11614913
37	N. Wang [57]	2014	Asian	ESCC	PB	PCR-LDR	597/597	rs11614913
38	Ni [58]	2015	Asian	endometrial/ovarian cancer	PB	PCR	141/100	rs11614913, rs3746444
39	Omrani [59]	2014	Asian	breast cancer	PB	PCR	236/203	rs11614913, rs3746444
40	P. Dikaiakos [60]	2015	Caucasian	colorectal cancer	PB	PCR–RFLP	157/299	rs11614913
41	P. Li [61]	2014	Asian	Nasopharyngeal Carcinoma	PB	RT-PCR	1020/1006	rs11614913
42	P. Qi [62]	2015	Asian	breast cancer	PB	PCR–RFLP	321/290	rs11614913, rs3746444
43	Panagiotis [63]	2014	Caucasian	gastric cancer	HB	PCR–RFLP	163/480	rs11614913
44	Pavlakis [64]	2013	Caucasian	pancreatic cancer	HB	PCR–RFLP	93/122	rs11614913
45	Peng [65]	2010	Asian	gastric cancer	HB	PCR–RFLP	231/213	rs11614913
46	Qi [66]	2011	Asian	HCC	PB	PCR–LDR	361/391	rs11614913
47	Qu [67]	2014	Asian	ESCC	HB	PCR–RFLP	381/426	rs11614913
48	Rama [68]	2010	Asian	Bladder Cancer	PB	PCR–RFLP	212/250	rs11614913, rs3746444
49	Renata [69]	2012	Caucasian	colorectal cancer	PB	PCR–RFLP	197/212	rs11614913
50	Roshni [70]	2014	Asian	oral cancer	PB	PCR–RFLP	451/452	rs11614913
51	Serena [71]	2011	Caucasian	Lung Cancer	PB	RT-PCR	101/129	rs11614913, rs3746444
52	Shen [72]	2015	Asian	ESCC	PB	Hapmap	1400/2185	rs11614913, rs3746444
53	Sushma [73]	2015	Caucasian	Oral Squamous Cell Carcinoma	PB	PCR–RFLP	100/102	rs11614913, rs3746444
54	Tian [74]	2009	Asian	lung cancer	PB	PCR–RFLP	1058/1035	rs11614913, rs3746444
55	Wang [75]	2014	Asian	HCC	PB	PCR–RFLP	152/304	rs3746444
56	Wu [76]	2013	Asian	gastric cancer	PB	PCR–RFLP	200/211	rs3746444
57	Yan [77]	2015	Asian	HCC	PB	PCR–RFLP	274/328	rs11614913, rs3746444
58	Z.Hu [78]	2008	Asian	breast cancer	PB	PCR–RFLP	1009/1093	rs11614913, rs3746444
59	Zhang [79]	2013	Asian	Acute lymphoblastic leukemia	PB	TaqMan	570/673	rs11614913
60	Zhao [80]	2013	Asian	HCC	PB	PCR–RFLP	235/281	rs11614913, rs3746444
61	Zhou [81]	2014	Asian	HCC	PB	PCR–RFLP	266/281	rs11614913, rs3746444
62	Zhu [82]	2011	Asian	Colorectal Cancer	HB	RT-PCR	573/588	rs11614913

HB, hospital based; PB, population based; HCC, hepatocellular carcinoma; ESCC, esophageal squamous cell carcinomar; PCR-RFLP, polymerase chain reaction–restriction fragment length.

polymorphism; PCR-CTPP, polymerase chain reaction with confronting two-pair primers; LDR, ligation detection reaction.

### Statistical analysis

We first assessed the departure of frequencies of miRNA polymorphisms from expectation under Hardy-Weinberg equilibrium (HWE) for each study by using the goodness-of-fit test (chisquare or Fisher exact test) in controls. ORs corresponding to 95% CIs were calculated to access the strength of association between microRNA SNPs and cancer risks. Pooled ORs were obtained from combination of single study by heterozygote comparison (CT vs. CC for rs11614913; AG vs. AA for rs3746444), homozygote comparison (TT vs. CC for rs11614913; GG vs. AA for rs3746444), dominant model (TT + TC vs. CC for rs11614913; GG + AG vs. AA for rs3746444), recessive model (TT vs. CC + CT for rs11614913; GG vs. AG + AA for rs3746444) and allelic model (T vs. C for rs11614913; G vs. A for rs3746444) respectively. For each genetic comparison model, subgroup analysis according to ethnicity was investigated to estimate ethnic-specific ORs for Asian and Caucasian. Meanwhile stratified analyses by tumor type or control characteristics were also applied for each genetic comparison model.

Statistical heterogeneity between studies was checked by Cocharan’s chi-square based *Q*-test [18] and quantified by I^2^. If the *P*-value for heterogeneity was < 0.05, or if I^2^ was ≥ 50%, indicating substantial heterogeneity among studies, then a random-effect model using the DerSimonian and Laird method [31], which yielded wider CIs, was chosen to calculate the pooled OR; otherwise, a fixed-effect model using the Mantel-Haenszel method [19] was used. One-way sensitivity analyses were performed to assess the stability of the meta-analysis results [20]. Potential publication bias was estimated using Egger’s linear regression test by visual inspection of the Funnel plot. All *P* value < 0.05 was used as an indication of potential publication bias [21].

All statistical analyses were carried out with the review manager version 5.2 (Revman; The Cochrane Collaboration, Oxford, UK). All *P* values in the meta-analysis were two-sided, and *P* value less than 0.05 were considered significant.

## RESULTS

### Characteristics of the studies

**Figure 1:**
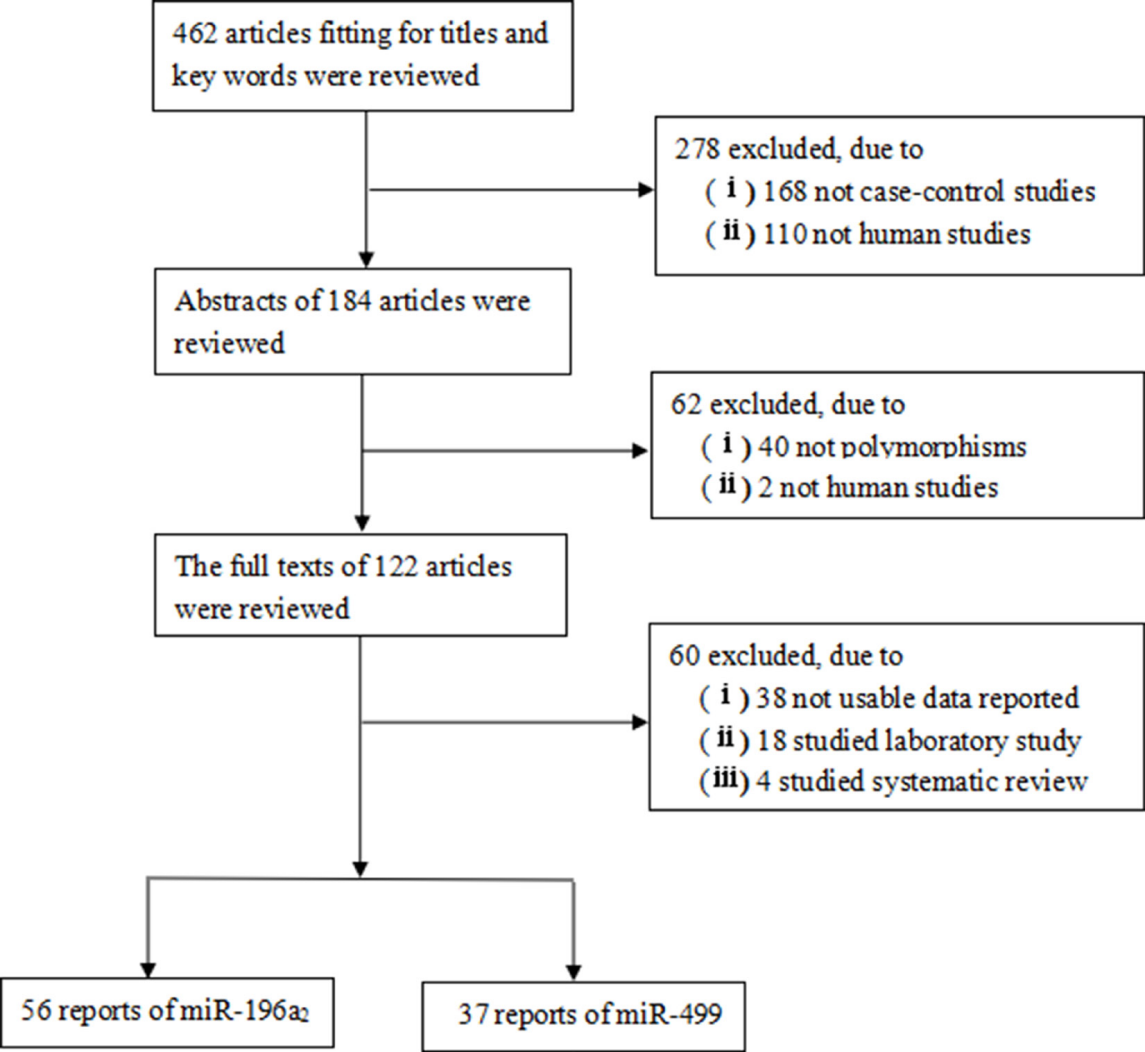
Flow chart of the study selection process

In total, 462 published studies were obtained though literature search, including the PubMed, EMBASE and CNKI database. Under conditions prescribed by the inclusion and exclusion criteria, 122 eligible studies (Figure 1) were retrieved, because they were no detailed evaluation. During data extraction, 62 eligible studies [22–83] were leaved, in which 56 and 37 studies were pooled for our meta-analysis, respectively (Figure 1). The characteristics of these selected studies are summarized in Table 1. Among all the included studies, there were 13 studies (hepatocellular cancer), 12 studies (breast), 7 studies (gastric), 6 studies (colorectal), 4 studies (lung), and 20 studies (other cancer types), and one (breast/ovarian cancer). There were 48 studies of Asian population, 14 studies of Caucasian population. Generally speaking, 56 studies included in our meta-analysis with 21958 cases and 26436 controls, which were ultimately analyzed for miR-196a2C.T (rs11614913), 37 studies including 13759 cases and 17946 controls for miR-499 A.G(rs3746444) .

### Quantitative synthesis

#### miR-196a2C.T (rs11614913)

For miR-196a2C.T rs11614913 polymorphism, our mate-analysis contain 56 studies (21958 cases and 26436 controls). We observed the T allele frequency via different ethnicities (Asian: 0.93, 95% CI = 0.91–0.96; Caucasian: 0.96, 95% CI = 0.90–1.02).

In the overall analysis, our mate-analysis results manifested a statistically significant association between the miR-196a2C.T rs11614913 and the reduced risks of cancers (OR = 0.93, 95% CI = 0.91–0.96, P_H_ < 0.00001 for T vs. C), homozygote comparison (OR = 0.88, 95% CI = 0.83–0.93, P_H_ < 0.00001 for TT vs. CC), dominant model (OR = 0.92, 95% CI = 0.89–0.96, P_H_ < 0.00001 for TT + CT vs. CC) and recessive model (OR = 0.94, 95% CI = 0.90–0.98, P_H_ < 0.00001 for TT vs. CC + CT ) (Supplementary Table 1).

**Figure 2:**
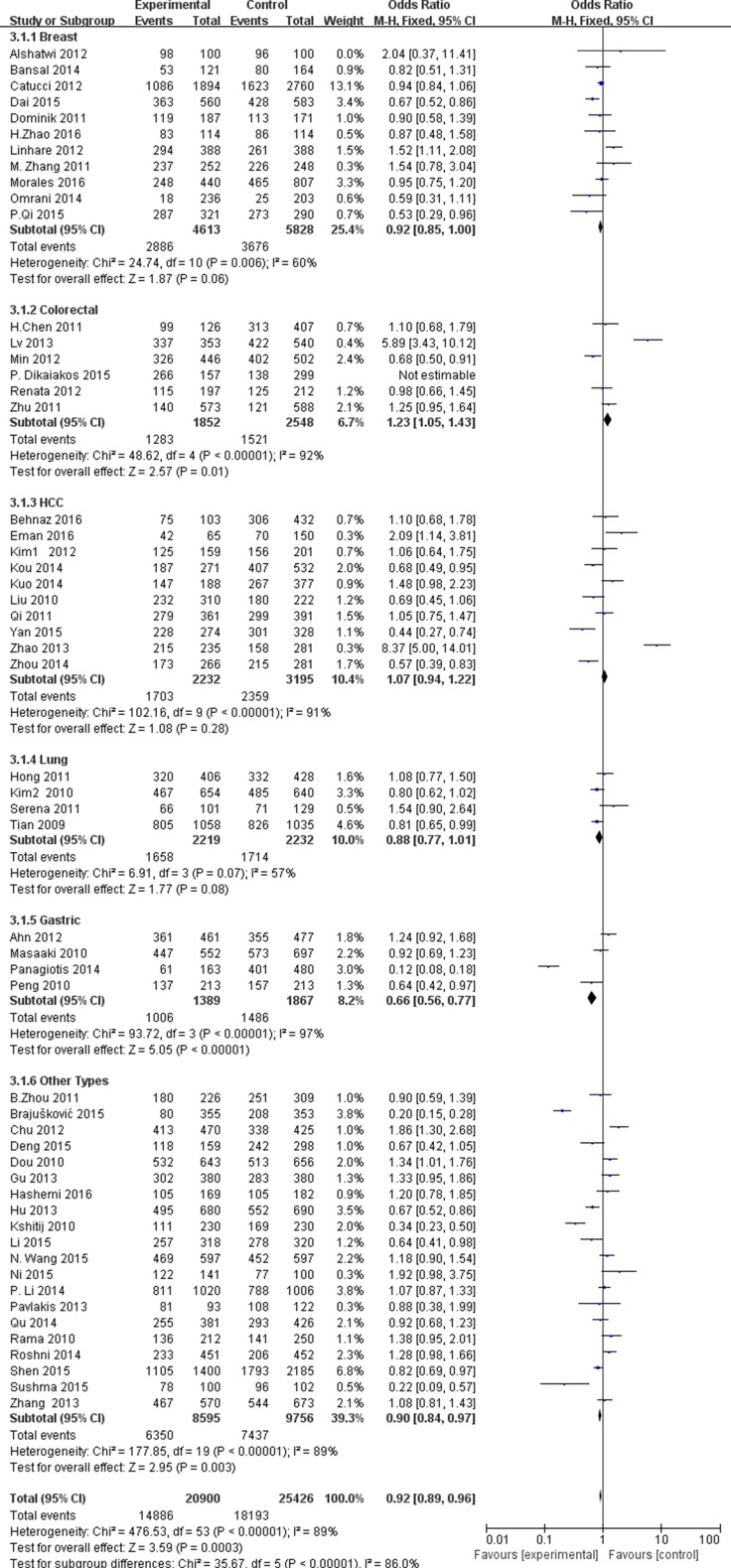
Forest plot of cancer risk in different cancer types associated with miR-196a2 rs11614913 polymorphism for recessive model (TT + TC vs. CC)

**Figure 3:**
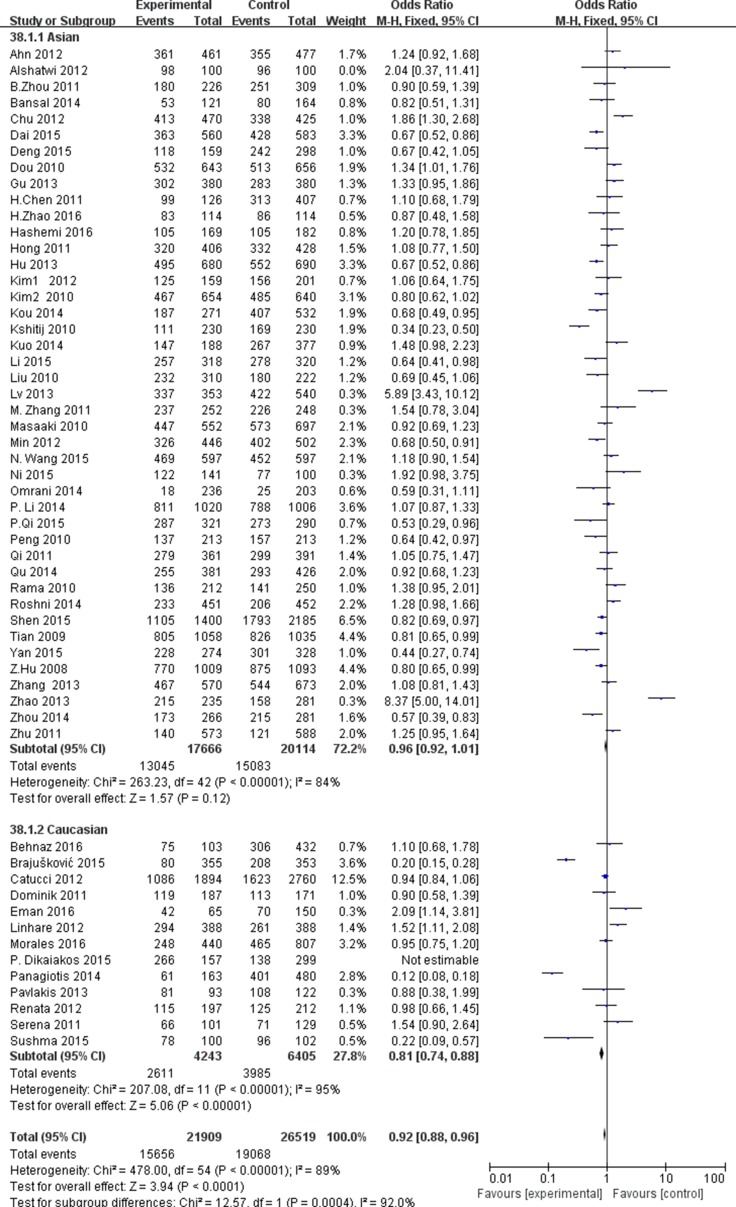
Forest plot of cancer risk in different ethnicity associated with miR-196a2 rs11614913 polymorphism for recessive model (TT+TC vs. CC)

In subgroup analysis by cancer types, we found the significant associations between the miR-196a2C.T rs11614913 and colorectal cancer (OR = 1.21, 95% CI = 1.11–1.33, P_H_ < 0.00001 for T vs. C; OR = 1.45, 95% CI = 1.21–1.74, P_H_ < 0.00001 for TT vs.CC; OR = 1.25, 95% CI = 1.06–1.46, P_H_ < 0.00001 for CT vs. CC; OR = 1.23, 95% CI = 1.05–1.43, P_H_ < 0.00001 for TT + TC vs. CC; OR = 1.72, 95% CI = 1.50–1.98, P_H_ < 0.00001 for TT vs. CC/TC); lung cancer (OR = 0.89, 95% CI = 0.82–0.97, P_H_ = 0.008 for T vs.C; OR = 0.79, 95% CI = 0.67–0.94, P_H_ = 0.26 for TT vs.CC; OR = 0.84, 95% CI = 0.74–0.96, P_H_ = 0.2 for TT vs. CC + TC); gastric cancer (OR = 0.77, 95% CI = 0.69–0.85, P_H_< 0.00001 for T vs. C; OR = 0.54, 95% CI = 0.45–0.66, P_H_ < 0.00001 for TT vs. CC; OR = 0.63, 95% CI = 0.52–0.75, P_H_ < 0.00001 for CT vs. CC; OR = 0.66, 95% CI = 0.56–0.77, P_H_ < 0.00001 for TT + CT vs. CC; OR = 0.76, 95% CI = 0.65–0.89, P_H_ < 0.00001 for TT vs. CC + TC ). In addition, we also found the decreased risks in other cancer types (OR = 0.86, 95% CI = 0.82–0.90, P_H_ < 0.00001 for T vs. C; OR = 0.90, 95% CI = 0.83–0.98, P_H_ < 0.00001 for TT vs. CC; OR = 0.90, 95% CI = 0.84–0.97, P_H_ < 0.00001 for TT + CT vs. CC; OR = 0.87, 95% CI = 0.81–0.93, P_H_ < 0.00001 for TT vs. CC + TC ) (Figure 2). Subgroup analysis by the ethnicity revealed a significant association in the comparison of T vs.C (OR = 0.93, 95% CI = 0.91–0.96, P_H_ < 0.00001), TT vs. CC (OR = 0.87, 95% CI = 0.82–0.92, P_H_ < 0.00001), TT vs. CC + TC (OR = 0.91, 95% CI = 0.87–0.95, P_H_ < 0.00001) in the Asian (Figure 3). Subgroup analysis by the source of control indicated a decreased risk in hospital based study, as showed in Supplementary Table 1.

### miR-499 A.G rs3746444

For miR-499A.G rs3746444, our mate-analysis included 37 studies (13759 cases and 17946 controls). our mate-analysis results are showed in Supplementary Table 2. On the whole, we found that miR-499A.G rs3746444 was significantly associated with decreased risks of cancers under the G vs. A (OR = 1.14, 95% CI = 1.09–1.19, P_H_ < 0.00001), GG vs. AA (OR = 1.20, 95% CI = 1.08–3.11, P_H_ < 0.001), AG vs. AA (OR = 1.06, 95% CI = 1.01–1.11, P_H_ < 0.00001 for), GG + GA vs. AA (OR = 1.16, 95% CI = 1.08–1.25, P_H_ = 0.07) and GG vs. AG + AA (OR = 1.20, 95% CI = 1.09–1.33, P_H_ < 0.00001).

**Figure 4:**
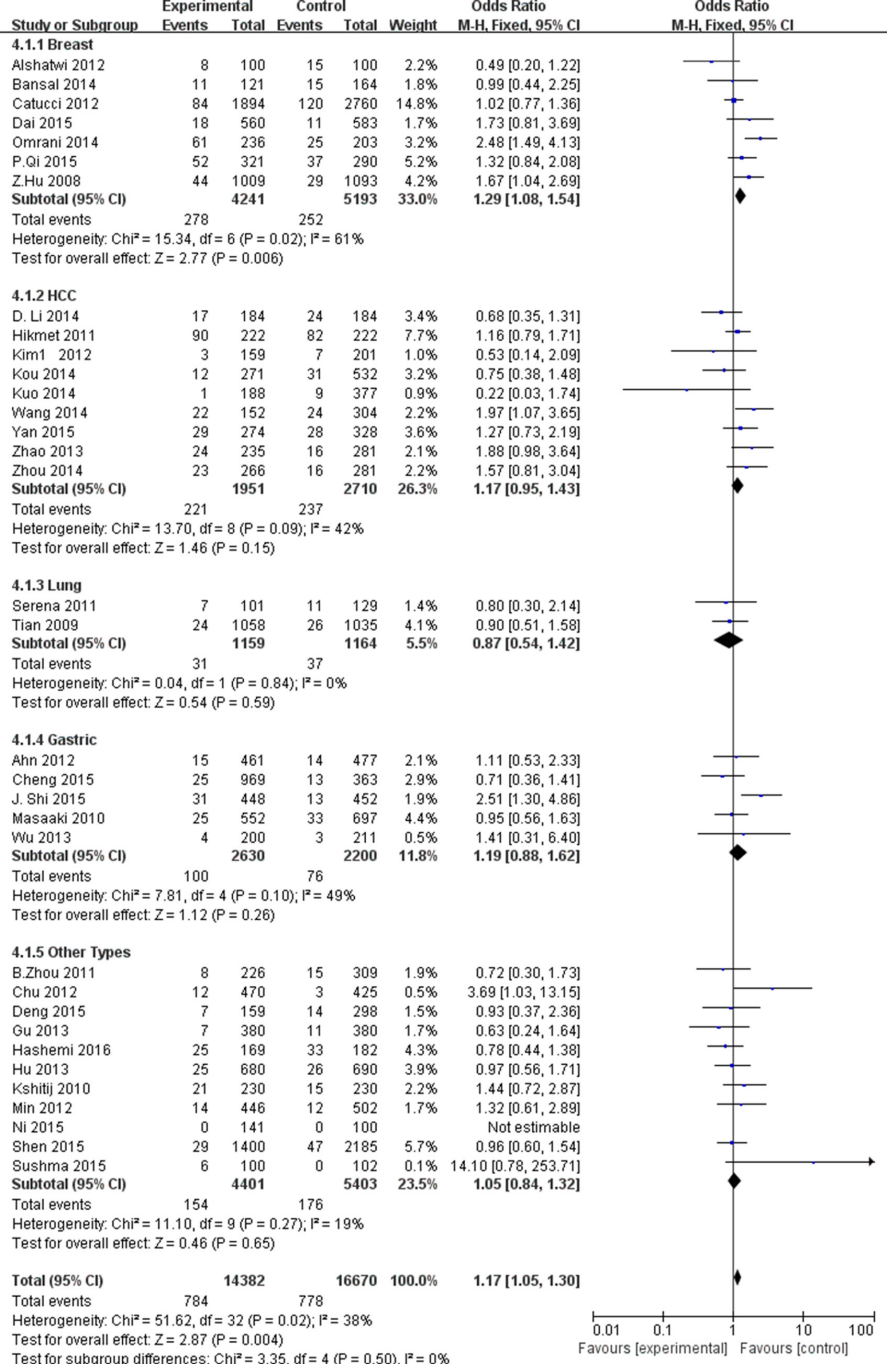
Forest plot of cancer risk in different cancer types associated with miR-499 rs3746444 polymorphism for recessive model (GG+GA vs. AA)

**Figure 5:**
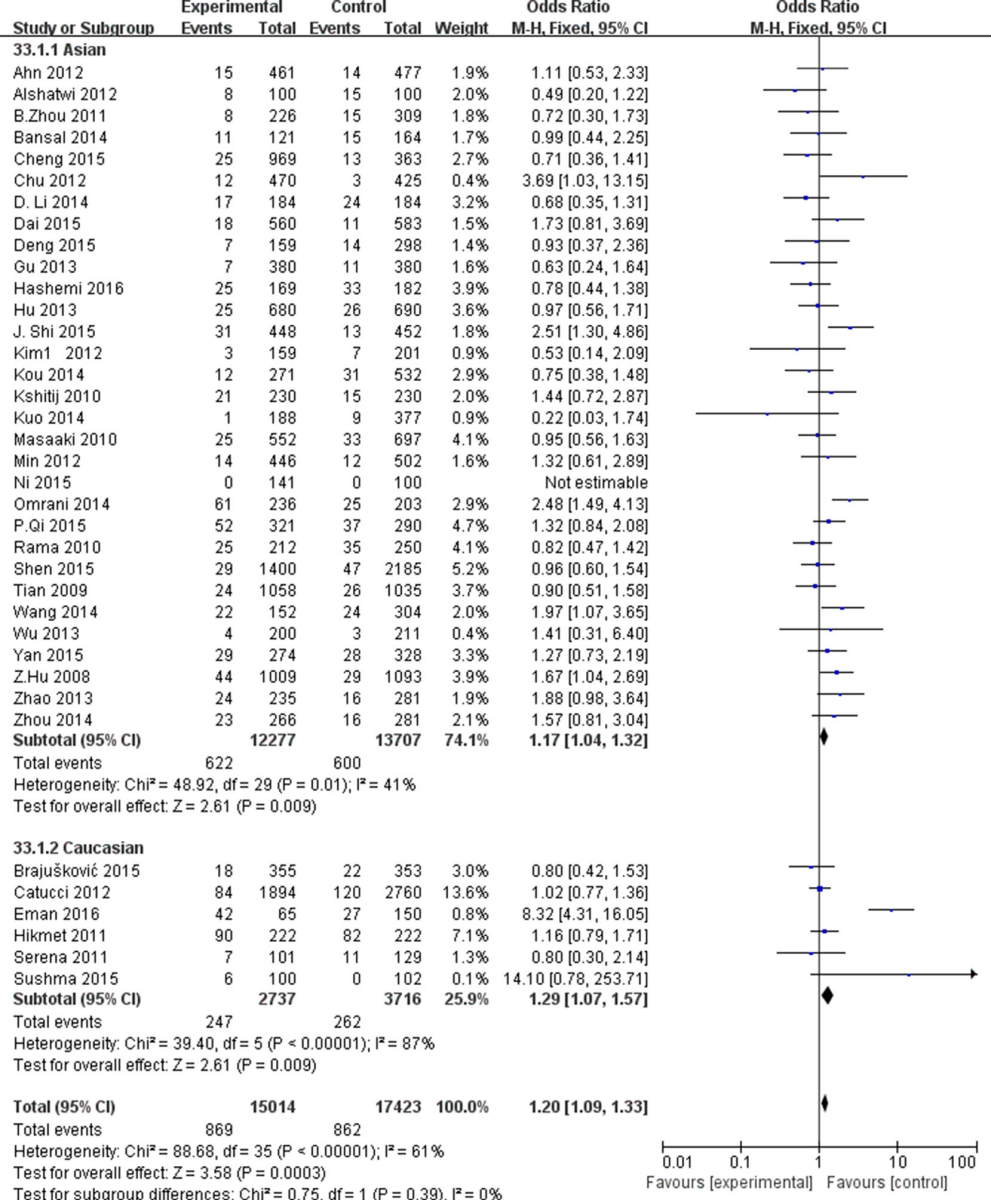
Forest plot of cancer risk in different ethnicity associated with miR-499 rs3746444 polymorphism for recessive model (GG+GA vs. AA)

In stratified analysis according to cancer types, we investigated the significant associations with breast cancer were only maintained under the G vs. A (OR = 1.18, 95% CI = 1.09–1.27, P_H_ = 0.04), GG vs. AA (OR = 1.29, 95% CI = 1.08–1.56, P_H_ = 0.04), GG + GA vs. AA (OR = 1.29, 95% CI = 1.08–1.54, P_H =_ 0.02 ) and GG vs. AG + AA (OR = 1.18, 95% CI = 1.08–1.29, P_H_ = 0.18). However, no statistically significant association was found in colorectal, lung, liver or other types cancers (Figure 4). Subgroup analysis according to ethnicity, significant associations with increased risks of cancers were found in Asian population (OR = 1.13, 95% CI = 1.08–1.19, P_H_ < 0.00001 for G vs. A; OR = 1.19, 95% CI = 1.06–1.34, P_H_ = 0.006 for GG vs. AA; OR = 1.17, 95% CI = 1.04–1.32, P_H =_ 0.01 for GG + GA vs. AA; OR = 1.12, 95% CI = 1.06–1.19, P_H_ < 0.00001 for GG vs. AG + AA), and in Caucasian (OR = 1.16, 95% CI = 1.06–1.26, P_H_ < 0.00001 for G vs. A; OR = 1.29, 95% CI = 1.07–1.57, P_H_ < 0.00001 for GG + GA vs. AA; OR = 1.16, 95% CI = 1.04–1.29, P _H_ = 0.001 for GG vs. AG + AA) (Figure 5). According to study design, we found significant association between population-based studies with elevated risks of cancer (OR = 1.15, 95% CI = 1.10–1.20, P_H_ < 0.00001 for G vs. A; OR = 1.22, 95% CI = 1.09–1.36, P _H_ < 0.00001 for GG vs. AA; OR = 1.14, 95% CI = 1.08–1.20, P_H_ < 0.00001 for GG + GA vs. AA; OR = 1.14, 95% CI = 1.08–1.20, P_H_ < 0.00001 for GG vs. AG + AA), but the hospital-based studies was not observed a significant association summarized in Supplementary Table 2.

### Sensitivity analysis

In the sensitivity analysis, each study involved in our meta-analysis was deleted the influence of the individual data on the coalescent ORs. The results of sensitivity analysis showed no obvious effects in overall population.

### Publication bias

**Figure 6:**
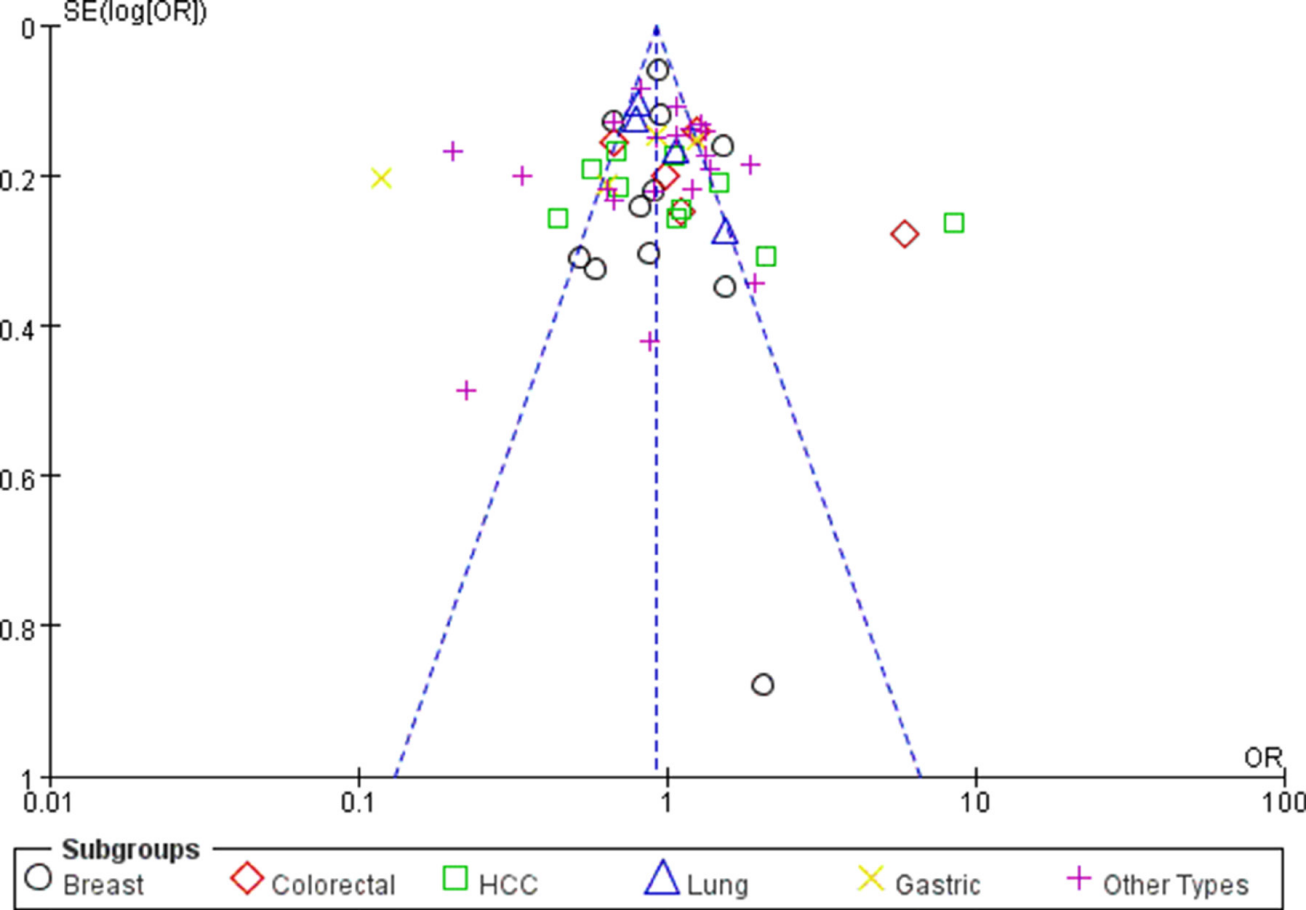
Funnel plot assessing evidence of publication bias (miR-196a2 rs11614913 (TT+TC vs. CC))

**Figure 7:**
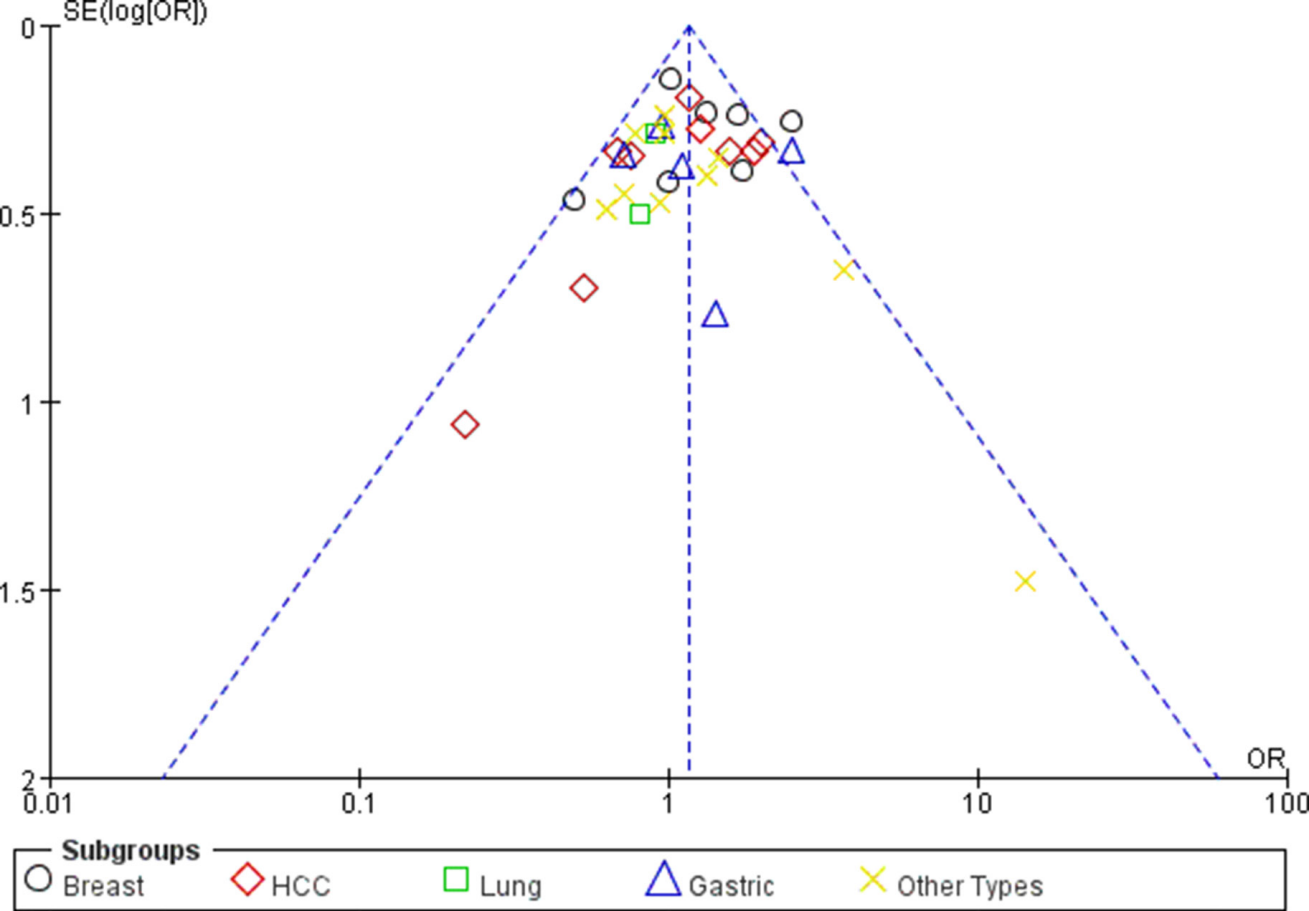
Funnel plot assessing evidence of publication bias (miR-499 rs3746444 (GG+GA vs. AA))

Begg’s funnel plot and Egger’s test were undertaken to evaluate the potential publication bias for this study. The shape of the Begg's funnel plots revealed no obvious asymmetry in all genotypes in overall population (Figures 6 and 7). The Egger’s test did not reveal publication bias (*P* > 0.05).

## DISCUSSION

The most common form of genetic sequence variation, SNPs are affecting miRNAs sequence coding, splicing and expression such as miRNA gene in human genome, which can affect the susceptibility of cancer including Asian and Caucasian [83]. It was detected in much previous research effort that the role of SNPs is located in miRNA sequence of miR-196a2 C.T (rs11614913) and miR-499 A.G (rs3746444) and influences on the progression of cancers [84]. Recently, several studies have investigated genetic variants of the miRNA SNPs in cancer susceptibility, but conclusions of those studies remain inconclusive. In this study, we conducted a meta-analysis to evaluated the association between the overall cancers susceptibility and the two polymorphisms in miRNA (miR-196a2 C.T rs11614913, miR-499 A.G rs3746444).

For miR-196a2 C.T rs11614913 polymorphism, although previous studies have revealed no association between cancer susceptibility and the expression of miR-196a2 rs11614913 [85–86]. Recently, the results of these meta-analysis studies have indicated a significant association between cancers susceptibility and miR-196a2 C.T rs11614913 [87–92]. The SNPs of miR-196a2 have caused increasing attention because they influence on the maturation progression and mutation of miRNA, and they play potential roles in tumor development and progression(cell proliferation, differentiation, apoptosis, migration and invasion). The predecessor found that the genetic sequence variation in miR-196a2 C.T rs11614913 is located in the 3′ passenger (3p) mature sequence of miR-196a2, and this functional polymorphism is reportedly associated with the susceptibility of multiple kind of tumors (lung cancer and breast cancer). However, there was lower survival rates in small cell lung cancer, gliomas, gastric cancer, gallbladder, head and neck, esophageal, and HCC . During this interim of more than one year, some relevant case-control studies have been published, while conclusions of the relevant studies remain incomprehensive and inconsistent. For example, Dai et al. [32] results revealed that the miR-499 A.G rs3746444 polymorphisms are related to increased risks of breast cancer, while the miR-196a2 C.T rs11614913 polymorphisms are connected with reduced risks of breast cancer. Wei’s studies [93] suggested that the miR-196a2 C.T rs11614913 might not be connected with susceptibility to gastric cancer, while our study revealed that the miR-196a2 C.T rs11614913 decrease risks to cancer, especially colorectal and gastric cancer in Asians population, and that the miR-499A.G rs3746444 may increase risks of cancer. In subgroup analysis by cancer type, ours results indicate that significant association with risks of cancer was observed in colorectal, gastric and lung cancer. But we did not detect significant association in breast cancer. While Christensen et al. [94] showed the miR-196a2C.T rs11614913 may reduced incidence of breast cancer. In subgroup analysis according to ethnicity, we found the significant association with risks of cancer in Asian population, indicating a possible role of differences in genetic backgrounds between Asians and Caucasians.

As for miR-499A.G rs3746444 polymorphism, the pooled results studies revealed that the miR-499A.G rs3746444 was association with risks of cancer in multiple types of cancer [95–97]. Several studies showed that a large amount of miRNAs are abnormally expressed in various cancers, and Zhang et al. [98] found that approximately 50% miRNA genes are located in cancer-associated regions, so miRNAs possibly exert a signifcant effect on the tumorigenesis. It was reported that studies have shown that the miR-499 rs3746444 can regulate the expression of SOX genes. The over-expression of SOX6 could reverse the anti-apoptosis effects of miR-499 A.G rs3746444 [99]. The abnormal expression of SOX genes can activate Wnt/β-catenin signaling pathway, which is associated with tumorigenesis and progression, so miR-499 A.G rs3746444 may play a decisive role in the occurring of cancer by altering SOX genes’ expression level. Moreover, the mate-analysis results from 37 studies revealed that the miR-499 rs3746444 G allele was revealed as a risk factor for cancers, in particular, for breast cancer or for in the Asian, which consistent with Hu’s results [100]. Therefore, The results illustrated that cancer types and district classification could cause different effects between miR-499 A.G rs3746444 polymorphism and risks of cancer.

To our knowledge, the source of the heterogeneity, including miR-196a2 C.T rs11614913 and miR-499A.G rs3746444, were mainly results from different ethnicity, different cancer types, different source of controls, different selection of subjects and sample size. Therefore, we evaluated the source of heterogeneity by cancer types, ethnicity, different selection of subjects and sample size. Nevertheless, our meta-analysis indeed exist some boundedness. Firstly, lack of relevant published data from the collected studies of potential gene-to-gene and gene-to-environment interactions, which may adjust risks of cancer. Secondly, potential heterogeneity was detected in some comparison, because they are unavoidable. Finally, publication bias existed in studies.

In conclusion, our meta-analysis indicated that the miR-196a2 C.T rs11614913 is significantly associated with a decreased risk of cancers, especially in the subgroup of colorectal, lung and gastric cancer, or Asians. Contrary to the above, the miR-499A.G rs3746444 most likely contributes to increased susceptibility of cancer in overall population, especially in breast cancer. Furthermore, more well-designed researches with large sample size are still necessary to elucidate the correlation between polymorphisms and different kinds of cancers risk.

## SUPPLEMENTARY MATERIALS TABLES






