# Supplementary material for: Association of miR-196a2 rs11614913 and miR-499 rs3746444 polymorphisms with cancer risk: a meta-analysis
Source: Oncotarget. 2017 Nov 20;8(69):114344–59. doi: 10.18632/oncotarget.22547 (PMC5768408; doi:10.18632/oncotarget.22547)
Supplement: Supplementary file 1 [file oncotarget-08-114344-s001.pdf]

Association of miR-196a2 rs11614913 and miR-499 rs3746444 polymorphisms with cancer risk: a meta-analysis

SUPPLEMENTARY MATERIALS

Supplementary Table 1: Meta-analysis of miR-196a2 rs11614913 polymorphism with cancer risk. See Supplementary_Table 1

Supplementary Table 2: Meta-analysis of miR-499 rs3746444 polymorphism with cancer risk. See Supplementary_Table_2
