# Supplementary material for: Association of miR-196a2 rs11614913 and miR-499 rs3746444 polymorphisms with cancer risk: a meta-analysis
Source: Oncotarget. 2017 Nov 20;8(69):114344–59. doi: 10.18632/oncotarget.22547 (PMC5768408; doi:10.18632/oncotarget.22547)
Supplement: Supplementary file 2 [file oncotarget-08-114344-s002.docx]

Supplementary Table 1: Meta-analysis of miR-196a2 rs11614913 polymorphism with cancer risk

| **Category** | **Cases/Controls** | **T vs. C** |  |  |  | **TT vs. CC** |  |  |  | **CT vs. CC** |  |  |  | **TT+CT vs. CC** |  |  |  | **TT vs. CC+CT** |  |  |  |
| --- | --- | --- | --- | --- | --- | --- | --- | --- | --- | --- | --- | --- | --- | --- | --- | --- | --- | --- | --- | --- | --- |
|  |  | **OR(95% CI)** | **P** | **P-H** | **I^2^** | **OR(95% CI)** | **P** | **P-H** | **I^2^** | **OR(95% CI)** | **P** | **P-H** | **I^2^** | **OR(95% CI)** | **P** | **P-H** | **I^2^** | **OR(95% CI)** | **P** | **P-H** | **I^2^** |
| **Total** | 21958/26436 | 0.93(0.91-0.96) | <0.00001 | <0.00001 | 88% | 0.88(0.83-0.93) | <0.00001 | <0.00001 | 82% | 0.97(0.93-1.02) | 0.27 | <0.00001 | 77% | 0.92(0.89-0.96) | <0.00001 | <0.00001 | 89% | 0.94(0.90-0.98) | 0.004 | <0.00001 | 85% |
| **Cancer types** |  |  |  |  |  |  |  |  |  |  |  |  |  |  |  |  |  |  |  |  |  |
| Breast cancer | 5611/6921 | 0.94(0.89-1.00) | 0.03 | <0.0001 | 73% | 0.90(0.80-1.02) | 0.11 | 0.001 | 68% | 0.95(0.87-1.04) | 0.03 | 0.1 | 38% | 0.92(0.85-1.00) | 0.06 | 0.006 | 60% | 0.90(0.81-1.00) | 0.05 | 0.007 | 61% |
| Colorectal cancer | 1852/2548 | 1.21(1.11-1.33) | <0.0001 | <0.00001 | 90% | 1.45(1.21-1.74) | <0.0001 | <0.00001 | 91% | 1.25(1.06-1.46) | 0.006 | <0.00001 | 89% | 1.23(1.05-1.43) | 0.01 | <0.00001 | 92% | 1.72(1.50-1.98) | <0.00001 | <0.00001 | 94% |
| HCC | 2274/3153 | 0.94(0.87-1.02) | 0.14 | <0.00001 | 91% | 0.83(0.70-0.97) | 0.02 | <0.00001 | 78% | 0.89(0.78-1.02) | 0.1 | 0.008 | 59% | 1.07(0.94-1.22) | 0.28 | <0.00001 | 91% | 0.90(0.78-1.04) | 0.15 | 0.002 | 68% |
| Lung cancer | 2219/2232 | 0.89(0.82-0.97) | 0.008 | 0.15 | 44% | 0.79(0.67-0.94) | 0.007 | 0.26 | 26% | 0.93(0.80-1.07) | 0.32 | 0.06 | 60% | 0.88(0.77-1.01) | 0.08 | 0.07 | 57% | 0.84(0.74-0.96) | 0.01 | 0.2 | 35% |
| Gastric cancer | 1407/1867 | 0.77(0.69-0.85) | <0.00001 | <0.00001 | 97% | 0.54(0.45-0.66) | <0.00001 | <0.00001 | 95% | 0.63(0.52-0.75) | <0.00001 | <0.00001 | 94% | 0.66(0.56-0.77) | <0.00001 | <0.00001 | 97% | 0.76(0.65-0.89) | 0.0008 | <0.00001 | 90% |
| Other cancers | 8501/9567 | 0.86(0.82-0.90) | <0.00001 | <0.00001 | 91% | 0.90(0.83-0.98) | 0.02 | <0.00001 | 76% | 1.07(0.99-1.15) | 0.09 | <0.00001 | 72% | 0.90(0.84-0.97) | 0.003 | <0.00001 | 89% | 0.87(0.81-0.93) | 0.0001 | <0.00001 | 85% |
| **Ethnicities** |  |  |  |  |  |  |  |  |  |  |  |  |  |  |  |  |  |  |  |  |  |
| Asian | 17715/20072 | 0.93(0.91-0.96) | <0.00001 | <0.00001 | 84% | 0.87(0.82-0.92) | <0.00001 | <0.00001 | 78% | 0.99(0.94-1.04) | 0.61 | <0.00001 | 72% | 0.96(0.92-1.01) | 0.12 | <0.00001 | 84% | 0.91(0.87-0.95) | <0.0001 | <0.00001 | 80% |
| Caucasian | 4243/6364 | 0.96(0.90-1.02) | 0.15 | <0.00001 | 94% | 0.85(0.75-0.96) | 0.007 | <0.00001 | 88% | 0.94(0.86-1.03) | 0.18 | <0.00001 | 87% | 0.81(0.74-0.88) | <0.00001 | <0.00001 | 95% | 1.06(0.96-1.18) | 0.24 | <0.00001 | 92% |
| **Design** |  |  |  |  |  |  |  |  |  |  |  |  |  |  |  |  |  |  |  |  |  |
| PB | 18321/22163 | 0.97(0.94-0.99) | 0.02 | <0.00001 | 86% | 0.93(0.88-0.99) | 0.02 | <0.00001 | 78% | 1.01(0.97-1.07) | 0.57 | <0.00001 | 70% | 0.97(0.93-1.02) | 0.21 | <0.00001 | 88% | 0.96(0.92-1.01) | 0.1 | <0.00001 | 85% |
| HB | 3637/4273 | 0.82(0.77-0.87) | <0.00001 | <0.00001 | 92% | 0.66(0.58-0.75) | <0.00001 | <0.00001 | 89% | 0.80(0.72-0.90) | <0.0001 | <0.00001 | 88% | 0.73(0.66-0.81) | <0.00001 | <0.00001 | 91% | 0.81(0.73-0.90) | <0.0001 | <0.00001 | 85% |

HCC, hepatocellular carcinoma; HB: hospital based; PB: population based; HWE: Hardy-Weinberg equilibrium; OR: odds ratio; CI: confidence interval; P: p value; PH: P value of Q for heterogeneity test; I2: 0–25%, no heterogeneity; 25–50%, modest heterogeneity; 50%, high heterogeneity; Random effects model was used when P value of Q for heterogeneity test (P-H) ,0.05 or I^2^.50%; otherwise, fixed effect model was used.
