# Supplementary material for: Association of miR-196a2 rs11614913 and miR-499 rs3746444 polymorphisms with cancer risk: a meta-analysis
Source: Oncotarget. 2017 Nov 20;8(69):114344–59. doi: 10.18632/oncotarget.22547 (PMC5768408; doi:10.18632/oncotarget.22547)
Supplement: Supplementary file 3 [file oncotarget-08-114344-s003.docx]

Supplementary Table 2: Meta-analysis of miR-499 rs3746444 polymorphism with cancer risk.

| **Category** | **Cases/Controls** | **G vs. A** |  |  |  | **GG vs. AA** |  |  |  | **AG vs. AA** |  |  |  | **GG+AG vs. AA** |  |  |  | **GG vs. AG+AA** |  |  |  |
| --- | --- | --- | --- | --- | --- | --- | --- | --- | --- | --- | --- | --- | --- | --- | --- | --- | --- | --- | --- | --- | --- |
|  |  | **OR(95% CI)** | **P** | **P-H** | **I^2^** | **OR(95% CI)** | **P** | **P-H** | **I2** | **OR(95% CI)** | **P** | **P-H** | **I2** | **OR(95% CI)** | **P** | **P-H** | **I2** | **OR(95% CI)** | **P** | **P-H** | **I2** |
| **Total** |  | 1.14(1.09-1.19) | <0.00001 | <0.00001 | 80% | 1.20(1.08-3.11) | 0.0006 | <0.001 | 59% | 1.06(1.01-1.11) | 0.03 | <0.00001 | 64% | 1.16(1.08-1.25) | <0.0001 | 0.07 | 28% | 1.20(1.09-1.33) | 0.0003 | <0.00001 | 61% |
| **Cancer types** |  |  |  |  |  |  |  |  |  |  |  |  |  |  |  |  |  |  |  |  |  |
| Breast cancer | 4230/5193 | 1.18(1.09-1.27) | <0.0001 | 0.04 | 55% | 1.29(1.08-1.56) | 0.0006 | 0.04 | 55% | 1.10(1.00-1.21) | 0.05 | 0.05 | 52% | 1.29(1.08-1.54) | 0.006 | 0.02 | 61% | 1.18(1.08-1.29) | 0.0002 | 0.18 | 33% |
| HCC | 2058/2818 | 1.07(0.97-1.19) | 0.19 | 0.009 | 61% | 1.11(0.89-1.38) | 0.37 | 0.005 | 64% | 0.87(0.76-1.00) | 0.06 | 0.009 | 61% | 1.17(0.95-1.43) | 0.15 | 0.09 | 42% | 1.08(0.95-1.23) | 0.23 | 0.01 | 58% |
| Lung cancer | 1159/1164 | 0.96(0.82-1.13) | 0.64 | 0.83 | 0% | 0.88(0.54-1.44) | 0.61 | 0.92 | 0% | 0.98(0.81-1.18) | 0.84 | 0.6 | 0% | 0.87(0.54-1.42) | 0.59 | 0.84 | 0% | 0.97(0.81-1.16) | 0.74 | 0.68 | 0% |
| Gastric cancer | 2024/2806 | 0.96(0.85-1.07) | 0.41 | 0.007 | 72% | 1.16(0.85-1.59) | 0.34 | 0.07 | 54% | 0.88(0.77-1.01) | 0.07 | 0.18 | 36% | 1.19(0.88-1.62) | 0.26 | 0.1 | 49% | 0.91(0.80-1.04) | 0.16 | 0.04 | 61% |
| Other cancers | 4428/5315 | 1.28(1.19-1.37) | <0.00001 | <0.00001 | 88% | 1.31(1.07-1.61) | 0.01 | <0.0001 | 71% | 1.20(1.10-1.32) | <0.0001 | 0.0003 | 67% | 1.05(0.84-1.32) | 0.65 | 0.27 | 19% | 1.26(1.04-1.54) | 0.02 | <0.00001 | 77% |
| **Ethnicities** |  |  |  |  |  |  |  |  |  |  |  |  |  |  |  |  |  |  |  |  |  |
| Asian | 11702/14271 | 1.13(1.08-1.19) | <0.00001 | <0.00001 | 76% | 1.19(1.06-1.34) | 0.005 | 0.006 | 44% | 1.06(1.00-1.12) | 0.06 | <0.00001 | 67% | 1.17(1.04-1.32) | 0.009 | 0.01 | 41% | 1.12(1.06-1.19) | <0.0001 | <0.00001 | 65% |
| Caucasian | 2737/3675 | 1.16(1.06-1.26) | 0.0009 | <0.00001 | 90% | 1.24(1.00-1.53) | 0.05 | <0.00001 | 85% | 1.06(0.95-1.20) | 0.3 | 0.2 | 32% | 1.29(1.07-1.57) | 0.009 | <0.00001 | 87% | 1.16(1.04-1.29) | 0.008 | 0.001 | 75% |
| **Design** |  |  |  |  |  |  |  |  |  |  |  |  |  |  |  |  |  |  |  |  |  |
| PB | 12836/15704 | 1.15(1.10-1.20) | <0.00001 | <0.00001 | 79% | 1.22(1.09-1.36) | 0.0004 | <0.00001 | 60% | 1.06(1.00-1.12) | 0.04 | <0.00001 | 62% | 1.14(1.08-1.20) | <0.00001 | <0.00001 | 64% | 1.14(1.08-1.20) | <0.00001 | <0.00001 | 64% |
| HB | 1603/2242 | 1.05(0.92-1.20) | 0.45 | 0.0003 | 88% | 1.05(0.72-1.53) | 0.8 | 0.14 | 50% | 1.06(0.91-1.24) | 0.43 | 0.001 | 85% | 1.03(0.71-1.50) | 0.87 | 0.23 | 32% | 1.06(0.92-1.23) | 0.42 | 0.0005 | 87% |

HCC, hepatocellular carcinoma; HB: hospital based; PB: population based; HWE: Hardy-Weinberg equilibrium; OR: odds ratio; CI: confidence interval; P: p value; P-H: P value of Q for heterogeneity test; I2: 0–25%, no heterogeneity; 25–50%, modest heterogeneity; 50%, high heterogeneity;Random effects model was used when P value of Q for heterogeneity test (P-H),0.05 or I2.50%; otherwise, fixed effect model was used.
